# Primary small lymphocytic lymphoma of the breast: a rare presentation of non-Hodgkin’s lymphoma

**DOI:** 10.1093/omcr/omae028

**Published:** 2024-04-25

**Authors:** Matthew Passeggiata, Geovanna Badaro, Hui Un Kim, Landry Umbu, Penelope Mashburn, Manju Nath

**Affiliations:** Western Reserve Health Education/NEOMED General Surgery Residency Program, Warren, OH, USA; American University of Antigua Medical School, St. Johns Antigua; American University of Antigua Medical School, St. Johns Antigua; Western Reserve Health Education/NEOMED General Surgery Residency Program, Warren, OH, USA; Department of General Surgery, Trumbull Regional Medical Center, Warren OH, USA; Department of Pathology, Trumbull Regional Medical Center, Warren OH, USA

## Abstract

Primary Small Lymphocytic Lymphoma of the breast is a rare presentation of Non-Hodgkin’s lymphoma. In this report, we present the case of primary small lymphocytic lymphoma of the breast in a 65-year-old female who presented with an abnormal breast ultrasound significant for a nodule of the right breast consistent with BI-RADS 4, indicating follow-up with ultrasound-guided biopsy for further diagnostic evaluation. The patient had no prior history of extramammary lymphoma or widespread disease. A sample of the breast mass was obtained via ultrasound-guided core needle biopsy and the pathology report revealed low-grade B-cell Lymphoma. After discussion with medical oncology and the explanation of risks, benefits and alternatives to surgery, a lumpectomy was performed, and the final pathology report of the mass revealed primary low-grade B-cell lymphocytic lymphoma of the breast. On follow up, the PET scan was unremarkable and showed no evidence of abnormal glucose metabolism or adenopathy.

## INTRODUCTION

Breast lymphomas are classified into primary breast lymphoma (PBL) and secondary breast lymphoma (SBL) [1–3]. The incidence of PBL from 1975 to 2017 was 1.35/1 000 000 [4]. PBL is most commonly due to Non-Hodgkin’s Lymphoma (NHL) without previous diagnosis of extramammary lymphoma, whereas SBL is due to metastasis also most commonly from NHL that developed from extrammamary tissue. PBL and SBL are clinically similar, and both may exhibit B-symptoms such as fever, night sweats and weight loss [3]. NHL is a solid tumor of the lymphatic system, representing 90% of lymphomas [1]. NHL can be indolent or aggressive, Indolent NHL typically manifests with waxing and waning lymphadenopathy. Of the indolent types, the most common are follicular lymphoma, chronic lymphocytic leukemia (CLL), small lymphocytic lymphoma (SLL), and splenic marginal zone lymphoma [5].

## CASE PRESENTATION

**Figure 1:**
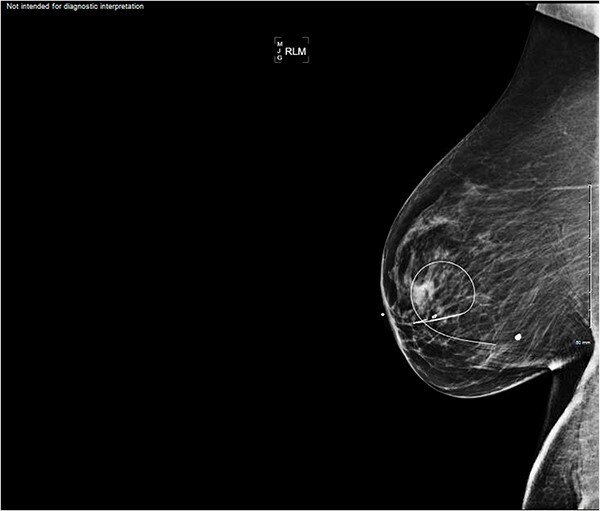
Imaging of the right breast mass at the 7 o’clock position measuring 4.6 × 3.5 × 3.6 cm.

An asymptomatic 65-year-old postmenopausal female with a past medical history of hypertension, diabetes mellitus, hyperlipidemia, and hysterectomy was referred to the surgery clinic after a non-diagnostic mammogram (BI-RADS Category 1) and ultrasound (US) significant for a nodule at the 7 o’clock position of the right breast measuring 4.6 × 3.5 × 3.6 centimeters (cm) (Fig. 1), consistent with BI-RADS 4, indicating the need for an US-guided biopsy for further diagnostic evaluation.

Her prior routine mammograms had been unremarkable for the past 6 years. Family history was significant for breast cancer in her sister who succumbed to aggressive metastatic breast cancer with unknown hormonal or HER2 status. The patient denied breast pain, nipple discharge, fever, night sweats, fatigue, chest pain, or palpitations. On exam there were no dimpling, retraction, or secretions of bilateral breasts. On palpation there was a palpable mass at the 7 o’clock position. There was no cervical, supraclavicular, or axillary lymphadenopathy.

The US-guided core needle biopsy revealed low-grade mature small B-cell lymphoma. The peripheral blood smear was within normal limits and complete blood count was normal outside of rare target cells, slight poikilocytosis, and rare schistocytes. There were no immature white blood cells and platelet count was normal with rare giant forms. The results were discussed with oncology, lumpectomy was recommended with follow up positron emission tomography (PET) scan imaging. After the explanation of risks, benefits, and alternative treatment options to lumpectomy the patient agreed to lumpectomy.

**Figure 2:**
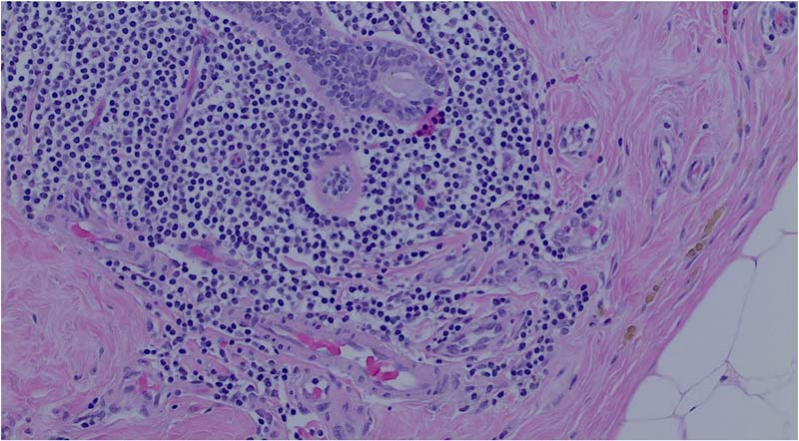
Atypical lymphoid cell population infiltrating the breast duct.

**Figure 3:**
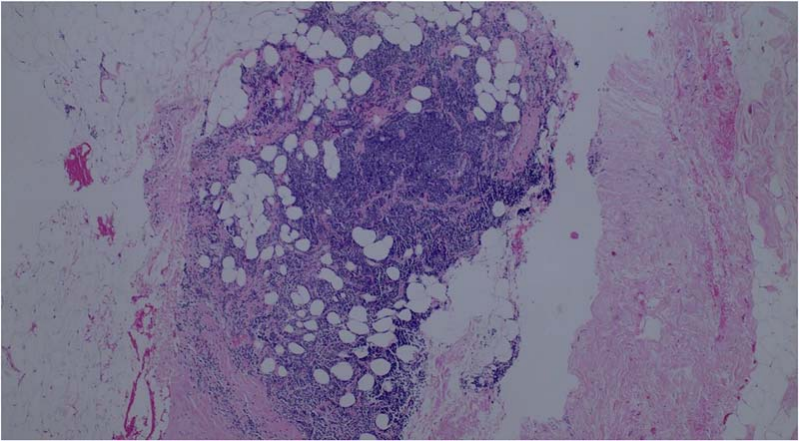
Lymphoid tissue infiltrating fat.

**Figure 4:**
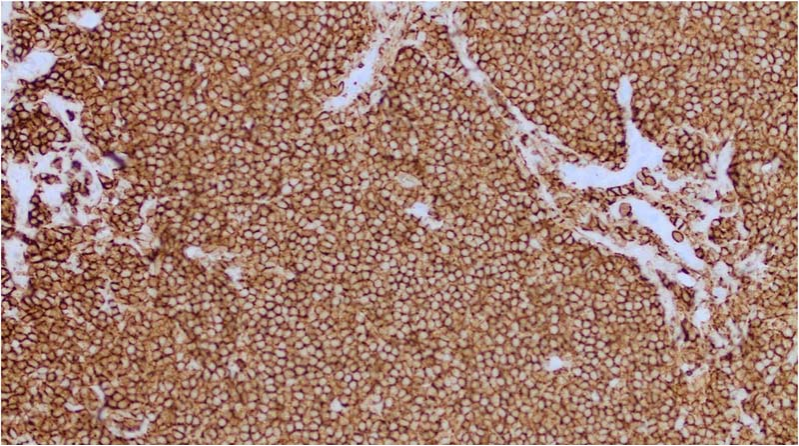
Immunohistochemical staining positive for B-cell marker CD20.

A lumpectomy was performed with no complications and the pathology report of the mass revealed atypical lymphoid cell population infiltrating the breast duct (Fig. 2) and lymphoid tissue infiltration fat (Fig. 3). Immunohistochemical staining confirmed low-grade NHL with positive B-cell markers for CD3, CD5, and CD20 (Fig. 4) and negative CD10, cyclin D1, BCL6, and CD23 markers. The final diagnosis of primary small lymphocytic lymphoma was made. On follow up, the PET scan was unremarkable with no evidence of abnormal glucose metabolism or adenopathy. The planned surveillance for this patient consisted of follow up mammogram imaging every 6 months for 1 to 2 years with yearly mammograms subsequently. A 6-month follow-up mammogram revealed no evidence of malignancy or significant changes.

## DISCUSSION

NHL is a heterogeneous group of lymphoproliferative malignancies originating from B and T-cell precursors, as well as mature B and T-cells [5]. 25% of NHL cases disseminate to extranodal areas, most commonly the stomach, Waldeyer’s ring, central nervous system, lung, bone, and skin [1]. Breast lymphoma is rare with a prevalence of 0.04%–0.7% [2, 6], which can be attributed to the scarcity of lymphoid tissue in the breast [4]. Breast lymphomas are classified into primary breast lymphoma (PBL) and secondary breast lymphoma (SBL) [1–3]. The current diagnostic criteria for PBL was initially proposed by Wiseman and Laio in 1972 [7]. The criteria consists of mammary tissue and lymphoma infiltrates in close proximity to each other, no evidence of concurrent widespread disease, and no prior diagnosis of extramammary lymphoma.

SLL consists of monomorphic small round B lymphocytes involving the lymph nodes, peripheral blood, and bone marrow [8]. In this patient, the histopathologic report of the mass showed two small foci of lymphoid infiltration predominantly composed of small lymphocytes positive for CD20, CD3, and CD5 markers. The peripheral blood smear showed a normal CBC without the presence of small lymphocytes. The presence of the neoplastic lymphocytes primarily in the lymphatic tissue rather than circulating in peripheral blood is more consistent with SLL rather than CLL [8].

PBL management consists of surgery, chemotherapy such as cyclophosphamide, hydroxy daunomycin, and vincristine sulfate, prednisone, radiotherapy, and immunotherapy, which can be done alone or in different combinations [9]. The classic treatment for PBL is chemotherapy, radical surgical excision has been shown to have no influence on survival or risk of recurrence therefore it is not recommended as a therapeutic measure [9, 10]. Despite this it is important to note that due to the scarcity of PBL, definite therapeutic guidelines have not been established. Therefore, surgical excision may be indicated when the core needle biopsy sample is insufficient for classification or due to individualized treatment plans and patient preference [10]. In this case, we have performed surgical excision for therapeutic purposes with follow-up PET scan dictating required additional treatment. If the PET scan detected metastasis of the PBL, then an assessment for further treatment would be followed.

## CONCLUSION

Primary breast lymphoma is a rare malignancy of the breast which shares many radiological and clinical presentations with breast carcinoma and other breast malignancies. Despite this, it is vital to have primary breast lymphoma as a differential diagnosis and to obtain an adequate sample for flow cytometry, allowing for an accurate diagnosis and appropriate treatment options. Although there are no definite therapeutic guidelines as there are for breast carcinoma, treatment options should be individualized to each patient’s case, taking stage, symptoms, overall health, patient preference and lymphoma subtype into consideration when deriving a treatment plan. Here we discussed the case of PBL successfully treated with surgical excision thereby avoiding the side effects and associated risks of chemotherapy and radiation.

## CONFLICT OF INTEREST STATEMENT

There are no conflicts of interests to disclose.

## CONSENT

Written informed consent was obtained for both the procedure and manuscript publication, including photographs and images.
